# Boosting Sensitivity in Liquid Chromatography–Fourier Transform Ion Cyclotron Resonance–Tandem Mass Spectrometry for Product Ion Analysis of Monoterpene Indole Alkaloids

**DOI:** 10.3389/fpls.2015.01127

**Published:** 2015-12-17

**Authors:** Ryo Nakabayashi, Hiroshi Tsugawa, Mariko Kitajima, Hiromitsu Takayama, Kazuki Saito

**Affiliations:** ^1^RIKEN Center for Sustainable Resource ScienceYokohama, Japan; ^2^Graduate School of Pharmaceutical Sciences, Chiba UniversityChiba, Japan

**Keywords:** metabolomics, monoterpene indole alkaloid (MIA), specialized metabolite, liquid chromatography–Fourier transform ion cyclotron resonance–tandem mass spectrometry, tandem mass spectrometry, MIAtargeted analysis

## Abstract

In metabolomics, the analysis of product ions in tandem mass spectrometry (MS/MS) is noteworthy to chemically assign structural information. However, the development of relevant analytical methods are less advanced. Here, we developed a method to boost sensitivity in liquid chromatography–Fourier transform ion cyclotron resonance–tandem mass spectrometry analysis (MS/MS boost analysis). To verify the MS/MS boost analysis, both quercetin and uniformly labeled ^13^C quercetin were analyzed, revealing that the origin of the product ions is not the instrument, but the analyzed compounds resulting in sensitive product ions. Next, we applied this method to the analysis of monoterpene indole alkaloids (MIAs). The comparative analyses of MIAs having indole basic skeleton (ajmalicine, catharanthine, hirsuteine, and hirsutine) and oxindole skeleton (formosanine, isoformosanine, pteropodine, isopteropodine, rhynchophylline, isorhynchophylline, and mitraphylline) identified 86 and 73 common monoisotopic ions, respectively. The comparative analyses of the three pairs of stereoisomers showed more than 170 common monoisotopic ions in each pair. This method was also applied to the targeted analysis of MIAs in *Catharanthus roseus* and *Uncaria rhynchophylla* to profile indole and oxindole compounds using the product ions. This analysis is suitable for chemically assigning features of the metabolite groups, which contributes to targeted metabolome analysis.

## Introduction

Liquid chromatography–mass spectrometry (LC–MS)-based metabolomics plays an important role in the interpretation of phytochemicals including primary and specialized metabolites (traditionally called secondary metabolites) in plants (Saito, 2013). The integrated analyses of LC–MS-based metabolomics and other omics such as genomics, transcriptomics, or proteomics, can be used to identify the biological gene-to-metabolite associations regarding these phytochemicals (Alseekh et al., 2015). Deciphering product ions possessing structural features in MS/MS analysis allows the precise targeted analysis of the phytochemicals. The identification of key product ions can help decipher basic skeleton (or aglycon) and modification parts of glycolipids (Okazaki et al., 2013; Tsugawa et al., 2015), glucosinolates (Sawada et al., 2009; Lee et al., 2012; Moussaieff et al., 2013), lignans (Morreel et al., 2004, 2014), flavonoids (Farag et al., 2007; Yonekura-Sakakibara et al., 2008), saponins (Pollier et al., 2011), and glycoalkaloids (Itkin et al., 2011; Iijima et al., 2013; Schwahn et al., 2014). The improvement of analytical methods focusing on chemical assignment of product ions in MS/MS analysis not only strongly affects the development of targeted analysis in LC–MS-based metabolomics but also facilitates remarkable achievements in applied studies such as synthetic biology, crop breeding, and phytochemical genomics.

Monoterpene indole alkaloids (MIAs) are one of the most important specialized metabolites in plants. Their complicated chemical structures exhibit a wide range of potent pharmaceutical activities such as anti-arrhythmic, anti-malarial, and anti-cancer effect (O’Connor and Maresh, 2006). Very recently, genes encoding biosynthetic enzymes involved in the biosynthetic pathways of MIAs in the medicinal plant *Catharanthus roseus* (Apocyanaceae), which includes a number of MIAs, have been identified in the study of phytochemical genomics (Besseau et al., 2013; Munkert et al., 2015; Stavrinides et al., 2015). Using identified biosynthetic genes, synthetic biological studies were performed to increase the production of strictosidine, which is the last common intermediate for all MIAs, in heterologous hosts (Miettinen et al., 2014; Brown et al., 2015; Qu et al., 2015).

Four methods are generally used for the profiling of MIAs. The first is thin layer chromatography (TLC) coupled with the Dragendorff’s color reagent. This method is used for checking whether N-containing metabolites exist in separated spots on a TLC plate. The second is ultraviolet (UV) spectrum-triggered LC. MIAs contain chromophore structures, which can be detected at approximately 254 nm. Tracing their UV spectrum enables profiling of compounds containing a chromophore. The third is mass-to-charge (*m/z*)-triggered LC–MS. MIAs are often profiled using the *m/z* value of precursor ions. The last method is a combination of the former ones. When many phytochemicals including alkaloids are detected in LC–MS analysis, all approaches are unfortunately inappropriate in terms of accuracy for the MIA-targeted analysis to seek known and unknown MIAs.

Product ions in MS/MS analysis are used in chemical assignments in LC–MS-based metabolomics. Among them, most monoisotopic ions have no chemical assignment of structural information, which suggests that there are unique ions contributing to the MIA-target analysis that have not yet been discovered. The ions can be used as the key to distinguish target metabolites from others. Boosting sensitivity in MS/MS analysis is more likely to highlight “hidden” product ions. To chemically assign “unearthed” product ions, both high accuracy and high peak resolution are required simultaneously. However, only a few methods exist to acquire extensive product ions at such high quality in MS/MS analysis. To address these points, the Fourier-transform ion cyclotron resonance–mass spectrometry (FTICR–MS) technique is one of the best tools, although cutting-edge quadrupole time-of-flight and Orbitrap MS instruments can routinely reach sub-ppm mass accuracy. In previous studies, FTICR–MS provided high-resolution product ions for the identification of their elemental composition (Nakabayashi et al., 2013, 2015a,b).

Here, we report a practical method to boost sensitivity in LC–FTICR–MS/MS analysis (i.e., MS/MS boost analysis) for chemically assigning elemental composition of extensive product ions. As a proof-of-concept, quercetin (^12^C quercetin, **1**) and uniformly labeled ^13^C quercetin (^13^C quercetin, **2**) were analyzed to verify the origin of the product ions is not the instrument, but the analyzed compounds resulting in sensitive product ions. The MS/MS boost analysis identified 100s of monoisotopic ions in MIAs having indole basic skeleton [ajmalicine (**3**), catharanthine (**4**), hirsuteine (**5**), and hirsutine (**6**)] and oxindole skeleton [formosanine (**7**), isoformosanine (**8**), pteropodine (**9**), isopteropodine (**10**), rhynchophylline (**11**), isorhynchophylline (**12**), and mitraphylline (**13**)]. The comparative analysis of the product ions showed common monoisotopic ions among the MIAs. Finally, this method was applied to the MIA-targeted analysis using the product ions. Compounds **3** and **4** in *C. roseus* and Compounds **11** and **12** in *Uncaria rhynchophylla* (Rubiaceae) were identified together with the candidates of indole and oxindole compounds, respectively. The results demonstrated that this analysis is suitable for chemically assigning features of the metabolite groups to product ions for LC–MS-based metabolomics.

## Materials and Methods

### Chemicals

Quercetin (^12^C quercetin, **1**) and uniformly labeled ^13^C quercetin (^13^C quercetin, **2**) were purchased from EXTRASYNTHESE (Genay, France) and SI Science (Saitama, Japan), respectively. Ajmalicine (**3**) and catharanthine (**4**) were purchased from Sigma Aldrich (Tokyo, Japan). Other alkaloids were isolated from plants that accumulate these compounds. The solutions of alkaloid compounds (100 μM) were prepared with EtOH (Supplementary Table S1).

### Plant Materials

Seeds of *C. roseus* “Equator White Eye” (Sakata Seeds, Co., Ltd., Kanagawa, Japan) were sown in the soil of pods [Pro-Mix BX (Premier Tech Horticulture, Inc., Rivière-du-Loup, QC, Canada): vermiculite = 2:1, supplemented with fertilizer]. The pods were set in a growth chamber at 24°C and a relative humidity of approximately 60% under a 16-h light (approximately 190 μmol s^-1^ m^-2^)/8-h dark cycle. The root parts of 6-weeks-old plants were harvested and immediately frozen in liquid nitrogen. The root samples were finally freeze-dried and stored at room temperature in a box containing silica gel. The hook parts of *U. rhynchophylla* were harvested in the medicinal plant garden of Chiba University (Chiba, Japan). The samples were dried and stored at room temperature in a box containing silica gel.

### Extraction of Metabolites

The samples were extracted with 50 μL of 80% MeOH per mg dry weight using a mixer mill (MM300, Retsch) with zirconia beads for 10 min at 20 Hz and 4°C. After centrifugation for 10 min, the supernatant was filtered using an HLB μElution plate (Waters).

### LC–FTICR–MS/MS Analysis

The solutions (5 μL) were analyzed using an LC–FTICR–MS instrument (LC, Agilent 1260 Infinity; MS, Bruker Daltonics SolariX 7.0 T). Analytical conditions were as follows. LC: column, Xselect CSH Phenyl-Hexyl (3.5 μm, 2.1 mm × 150 mm, Waters, Milford, MA, USA); solvent system, solvent A (water with 0.1% formic acid) and solvent B (acetonitrile with 0.1% formic acid); LC gradient program, 99.5% A/0.5% B at 0 min, 0.5% A/99.5% B at 30.0 min, 0.5% A/99.5% B at 45.0 min, 99.5% A/0.5% B at 45.1 min, and 99.5% A/0.5% B at 60.0 min; flow rate, 0.3 mL/min; column temperature, 35°C; wavelength, 200–600 nm; MS/MS detection; Acquisition Mass Control (mass range, *m/z* 100–600; estimated resolution power, 66,000 at *m/z* 400; transient length, 0.4893); Data Storage, save full profile spectrum, on; save FID file, on; Accumulation (average scan, 1; source accumulation, 0 s; ion accumulation time, 0.1 s; ion cooling time, 0 s; time of flight, 0.4 s); API Source [source type, electrospray ionization (ESI); capillary, 4500 V; end plate offset, -500 V]; Source Gas Tune (nebulizer 1.6 bar; dry gas, 7.0 L/min; dry temperature, 200°C); Source Optics (capillary exit, 220 V; deflector plate, 200 V; funnel 1, 150 V; skimmer 1, 25 V; funnel RF amplitude, 150 Vpp); Octopole (frequency, 5 MHz; RF amplitude, 350 Vpp); Collision Cell (collision voltage, -2 V; DC extract bias, 0.4 V polarity, RF frequency, 2 MHz; collision RF amplitude, 1200 Vpp); Transfer Optics (time of flight, 0.4 ms; frequency, 6 MHz, RF amplitude, 350 Vpp); Infinity Cell (transient exit lens, -20 V; analyzer entrance, -10 V; side kick, 0 V; side kick offset, -1.5 V; front trap plate, 0.6 V; back trap plate, 0.6 V; sweep excitation power, 18%); Multiple Cell Accumulations (number of ICR cell fills, 1); Gated Trapping (gated trapping mode, off); Precursor Selection (prefer list, *m/z* of target compound [M + H]^+^, mass accuracy, ±20 ppm; exclude unknown charged ions, on); Quadrupole Parameters (MS/MS isolation, on; cut off in MS, *m/z* 100; isolation window, *m/z* 5); Fragmentation Mode, quadrupole collision-induced dissociation (QCID); MS/MS Boost, on (in use); MS accumulation in MS/MS Boost, 0.1 s (in use); MS/MS accumulation in MS/MS Boost, 2 s (in use); fixed collision voltage, 30 V; polarity, positive; API high voltage, on; source quench, on.

### Data Analysis

The MS/MS spectra were recorded using Hystar 4.0 (Bruker Daltonik GmbH, Bremen, Germany) and the data were processed using DataAnalysis 4.2 (Bruker Daltonik GmbH). The analytical conditions of the MS/MS spectra were as follows: MS List: S/N threshold, 0; relative intensity threshold (base peak), 0%; absolute intensity threshold, 300,000; Chemistry: upper limit formula, the formula of [M + H]^+^; Charge, 1; relative intensity threshold, 1.0 × 10^-7^%, tolerance, ±2 mDa.

## Results and Discussion

**FIGURE 1:**
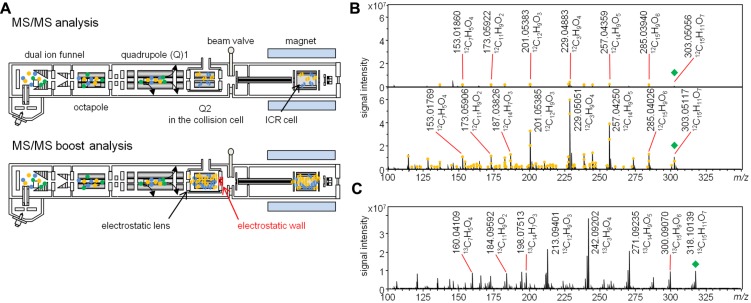
**Overview of the MS/MS boost analysis in the FTICR–MS instrument. (A)** Pattern diagram of the MS/MS and MS/MS boost analysis. **(B)** Comparison of the MS/MS analysis with the MS/MS boost analysis. Upper panel, MS/MS spectrum of quercetin (^12^C quercetin, **1**) in the MS/MS analysis; lower panel, MS/MS spectrum of Compound **1** in the MS/MS boost analysis. The yellow circle indicates the assigned product ion. The green diamond indicates the precursor ion. **(C)** MS/MS spectrum of uniformly ^13^C labeled quercetin (^13^C quercetin, **2**) in the MS/MS boost analysis. The green diamond indicates the precursor ion.

Here, we developed the MS/MS boost analysis method to boost the sensitivity in LC–FTICR–MS/MS analysis. The mechanism of the MS/MS boost analysis is as follows. Ions derived from the ESI source are detected in the ICR cell after going through dual ion funnels, octapole, and two quadrupoles in the FTICR–MS instrument. The first quadrupole selects appropriate ions, and the second one performs CID to generate product ions in the collision cell. The collection of precursor and product ions in the collision cell is facilitated by electrostatic lenses at the end of the collision cell. Ions are trapped in the collision cell by an electrostatic wall created by biasing the endplate to a high positive voltage. In this analysis, the accumulation of product ions leads to the increase of their concentration. After the trapping event, ions go to the ICR cell (**Figure 1A**) for the MS/MS boost analysis in the FTICR instrument. The MS/MS boost analysis is equipped in the FTICR instrument. The parameters of the MS/MS accumulation in the MS/MS Boost was set to accumulate product ions (see Materials and Methods). Here, the accumulation of product ions for 2 s enabled an increase in the sensitivity. There are general ways to boost the sensitivity in MS/MS analysis. One of them is, for example, to increase the sample concentration. However, excessive space of charge of the ion population leads to mass error in the FTICR instrument, which can be resolved by achieving low sample concentration. Another means is to perform direct infusion analysis in the FTICR instrument. Multiple scans dramatically gain sensitivity toward the product ions. However, this analysis induces complicated adduct ions, which cannot be used as reference ions for chemical assignment because most of those ions used in the chemical assignment are acquired using LC–MS/MS analysis. The use of the LC-hyphenated technology in the FTICR–MS/MS analysis enables separation of components in a sample and spreading of the ion population. It also enables acquiring simpler product ions, which makes it easy to understand structure information. Elemental compositions were assigned to monoisotopic ions of product ions on the basis of precursor ions as [M + H]^+^ with the software DataAnalysis, which calculates the formulae of monoisotopic ions using the exact mass and isotopic pattern.

To compare the signal intensity of the product ions in the MS/MS analysis (without setting the time to accumulate the product ions) with that of the product ions in the MS/MS boost analysis, flavonol quercetin was used in both analyses. The difference between the analyses was established by the degree of product ions accumulated. As a result, 28 ions of ^12^C quercetin (**1**) were detected at the retention time of 15.4 min in the MS/MS analysis. Elemental compositions were assigned to 14 monoisotopic ions (**Figure 1B**, upper panel). On the other hand, 197 ions of Compound **1** were detected in the MS/MS boost analysis; elemental compositions were assigned to 118 monoisotopic ions (**Figure 1B**, lower panel). The sensitivity of the base ion (*m/z* 229.04) increased approximately 20 fold in the MS/MS boost analysis (Dataset S1 in Supplementary Materials). To verify whether the product ions in the MS/MS boost analysis derived from the analyzed compounds, the product ions of Compound **1** were compared with those of ^13^C quercetin (Dataset S1 in Supplementary Materials). For the assigned ions, 98 ions of Compound **1** had corresponding product ions (mass error, ±2 mDa) in ^13^C quercetin (**2**; **Figure 1C**), indicating that the origin of the product ions could be assigned to the analyzed compounds.

**FIGURE 2:**
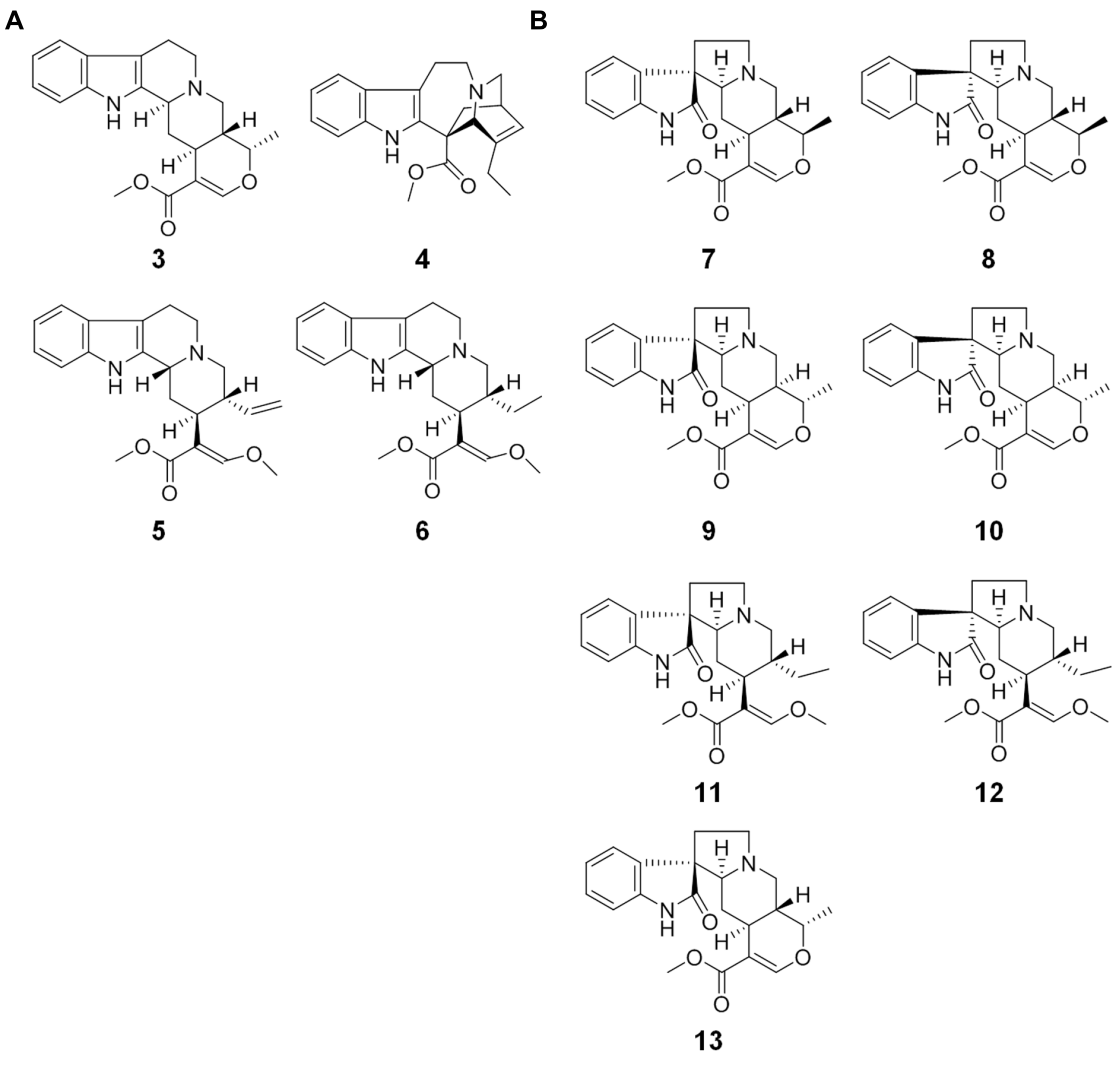
**Monoterpene indole alkaloids (MIAs) used in this study. (A)** MIAs having indole basic skeleton: **3**, ajmalicine; **4**, catharanthine; **5**, hirsuteine; **6**, hirsutine; **(B)** MIAs having oxindole basic skeleton: **7**, formosanine; **8**, isoformosanine; **9**, pteropodine; **10**, isopteropodine; **11**, rhynchophylline; **12**, isorhynchophylline; **13**, mitraphylline.

MS/MS analyses of MIAs have been previously reported, but the number of detailed product ion analysis is low for the MIA-targeted analysis in LC–MS-based metabolomics. In this study, 11 MIAs including four compounds having indole basic skeleton [ajmalicine (**3**), catharanthine (**4**), hirsuteine (**5**), and hirsutine (**6**); **Figure 2A**] and seven compounds having oxindole skeleton [formosanine (**7**), isoformosanine (**8**), pteropodine (**9**), isopteropodine (**10**), rhynchophylline (**11**), isorhynchophylline (**12**), and mitraphylline (**13**); **Figure 2B**] were investigated using the MS/MS boost analysis. Then, we performed comparative analyses using (i) the indole compounds, (ii) oxindole compounds, and (iii) three pairs of stereoisomers (**7** and **8**, **9** and **10**, and **11** and **12**) to reveal common product ions. Elemental compositions were extensively assigned to monoisotopic ions on the basis of CHNO. Single-charged monoisotopic ions were compared in the comparative analyses. When the monoisotopic ions had several candidates of elemental composition, the elemental composition with less mass error was considered.

**FIGURE 3:**
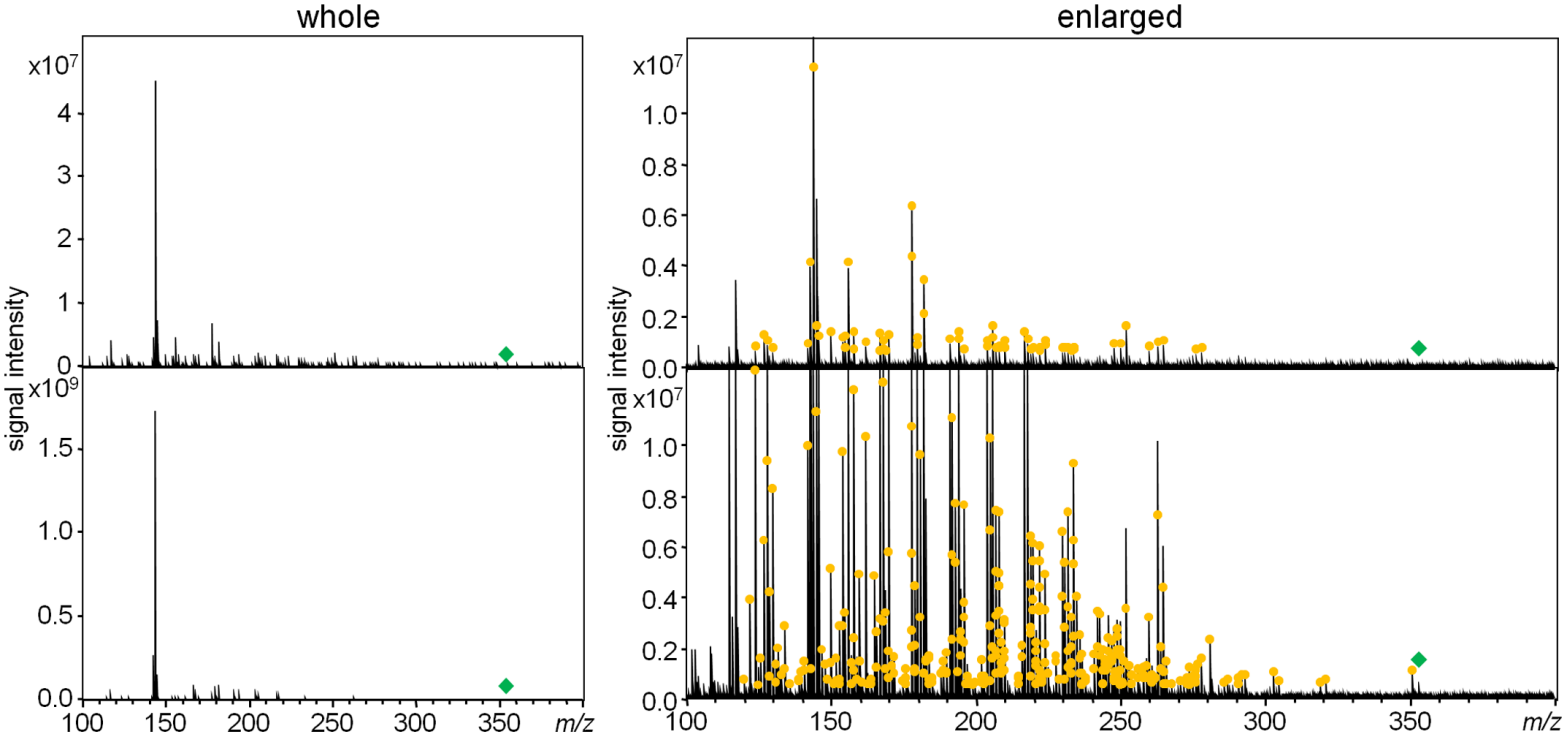
**Comparison of product ions of ajmalicine (**3**) in the MS/MS analysis **(upper)** and the MS/MS boost analysis (lower)**. The yellow circle indicates chemically assigned monoisotopic ions.

To acquire as many as possible product ions of the MIAs, MS/MS boost analysis at the collision energy of 30 V was performed. Collision energy values of 10, 30, and 50 V were preliminary considered using Compounds **3** and **4**. Ultimately, in this study, 30 V was selected as the best collision energy for extensively collecting product ions in less than the *m/z* values of their precursor ions. In the MS/MS boost analysis, on average, approximately fivefold more product ions were acquired than those acquired in the MS/MS analysis (**Table 1**). The fragmentation patterns of all the compounds in the MS/MS boost analysis were almost identical to those of the MS/MS analysis; however, a different number of product ions were obtained (**Figure 3** and Datasets S2–S5 in Supplementary Materials).

**Table 1:** Number of assigned monoisotopic ions in the MS/MS and MS/MS boost analysis.

Compound	Acquired ion		Assigned ion	
	MS/MS	MS/MS Boost	MS/MS	MS/MS Boost
3	82	540	73	345 (274)
4	34	301	24	190 (136)
5	338	874	236	504 (385)
6	180	876	143	487 (387)
7	91	552	67	342 (278)
8	128	525	100	335 (257)
9	243	771	155	440 (359)
10	135	696	96	412 (321)
11	62	440	47	276 (222)
12	179	672	125	403 (322)
13	120	545	79	348 (284)

*Number in parentheses is the total number of single charged ions*.

**FIGURE 4:**
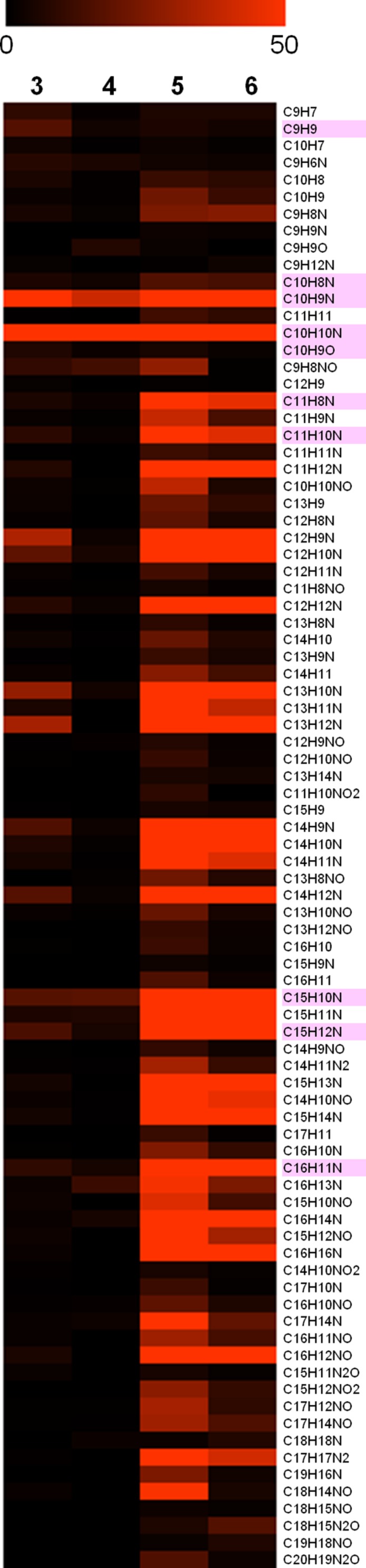
**Common product ions among the indole compounds (**3**–**6**) identified in the MS/MS boost analysis**. Heat map using relative signal intensities of the product ions. The color bar indicates 0–50% of relative signal intensity (the maximum is 1,000). The pink color indicates common ions of the indole compounds identified in the MS/MS analysis. The assigned elemental composition of the product ions is shown.

To identify common monoisotopic ions in the indole compounds (**Figure 2A**), the comparative analysis was performed on the basis of the assigned elemental composition. The result showed 86 common monoisotopic ions among the indole compounds (Dataset S6 in Supplementary Materials). The heat map indicates the variety of the signal intensity of the product ions, showing that the MS/MS boost analysis is useful for identifying major and minor product ions. The numbers of product ions assigned as CH, CHN, CHO, and CHNO were 14, 44, 2, and 26, respectively. The common ions of the indole compounds acquired in the MS/MS boost analysis were more than those found with the MS/MS analysis (**Figure 4**). The ion with the highest signal intensity (*m/z* 144.08, C_10_H_10_N) possibly derived from the substructure shown in Supplementary Figure S1. The ion was previously characterized as a key product ion of the indole skeleton (Lu et al., 2009). This result suggested that the analysis successfully assigned metabolite features of the indole compounds. The ions with lower signal intensity were acquired with good accuracy and reproducibility (Supplementary Figure S2).

**FIGURE 5:**
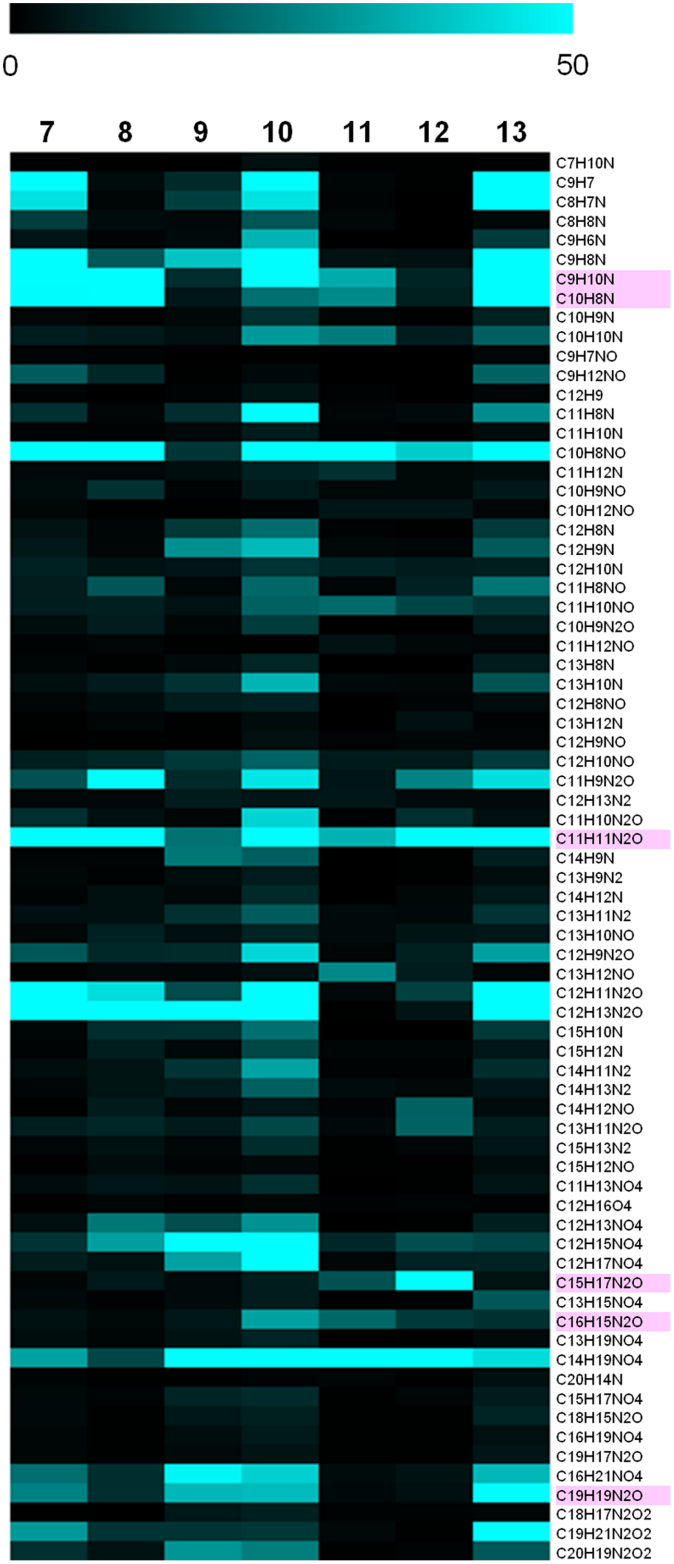
**Common product ions among the oxindole compounds (**7**–**13**) identified in the MS/MS boost analysis**. Heat map using relative signal intensities of the product ions. The color bar indicates 0–50% of relative signal intensity (the maximum is 1,000). The pink color indicates common ions of the oxindole compounds identified in the MS/MS analysis. The assigned elemental composition of the product ions is shown.

To identify common product ions in the oxindole compounds (**Figure 2B**), the comparative analysis was performed on the basis of the elemental composition. The result showed 73 common monoisotopic ions among the oxindole compounds (Dataset S7 in Supplementary Materials). The numbers of product ions assigned as CH, CHN, CHO, and CHNO were 2, 29, 1, and 41, respectively. Similar to the case of indole compounds, the common ions of the oxindole compounds acquired in the MS/MS boost analysis were more than those found with the MS/MS analysis (**Figure 5**). The ion (*m/z* 187.08, C_11_H_11_N_2_O) was possibly derived from the oxindole skeleton (Supplementary Figure S3). The ions with the lowest signal intensity in each compound are shown in Supplementary Figure S4.

The product ion analysis of stereoisomers in MIAs has not been fully performed in the context of metabolomics research. Comparative analyses of stereoisomers (**7** and **8**, **9** and **10**, and **11** and **12**) on the basis of the assigned elemental compositions were performed to identify common monoisotopic ions. These analyses showed that 185, 248, and 173 monoisotopic ions were common among each pair, respectively (Supplementary Figure S5 and Dataset S8–10 in Supplementary Materials). The common ions among each pair likely have different signal intensities (Supplementary Figures S3 and S6). Therefore, quantitative analysis of such signal intensities may show fragmentation mechanisms among the stereoisomers. The stereoisomer-specific ions could allow us to easily identify the stereoisomers in the chemical assignment of MIAs. Exploring alkaloid-specific ions requires much more comparative analyses using MIAs, which will be further investigated.

**FIGURE 6:**
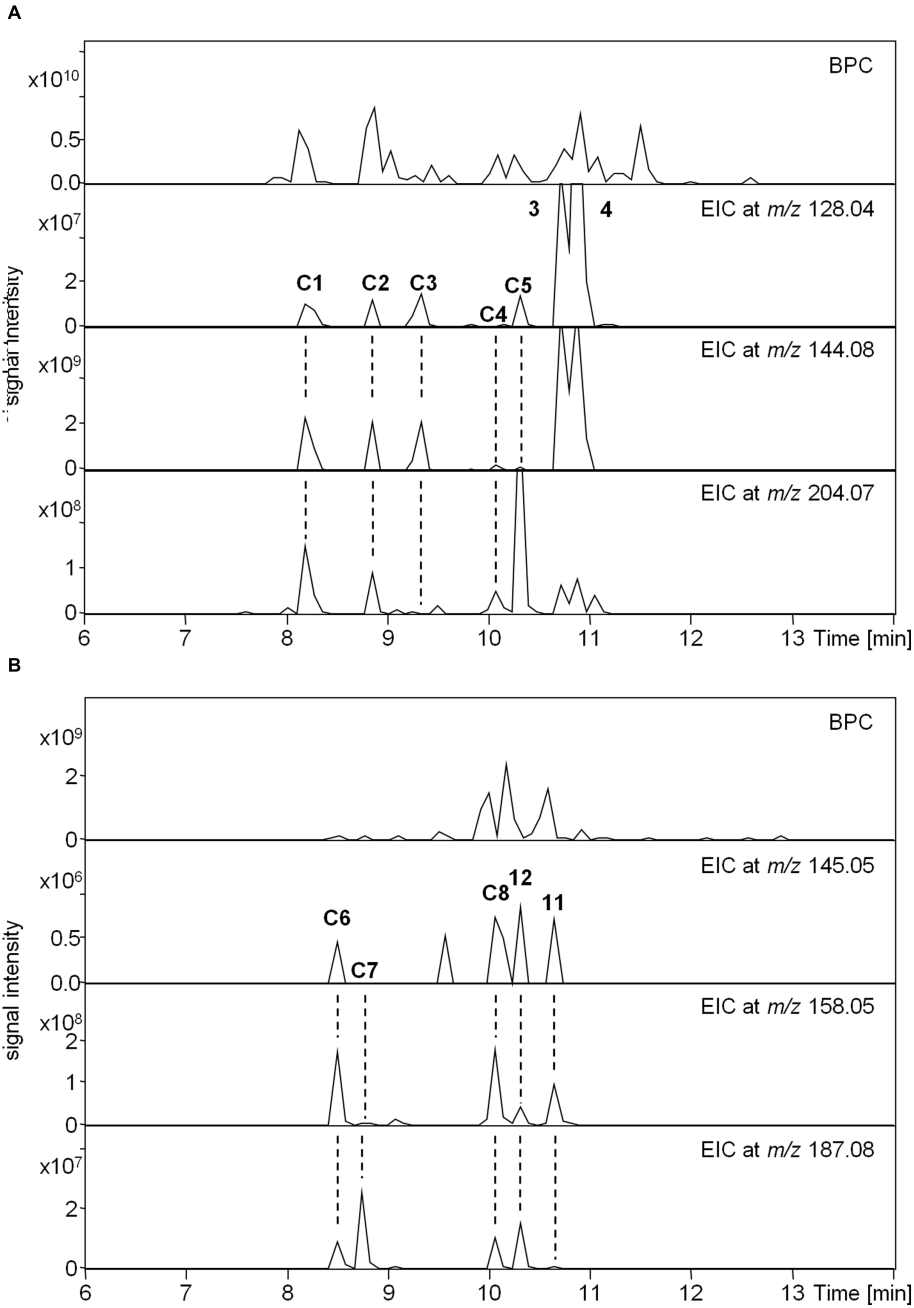
**Targeted metabolome analysis using the MS/MS boost analysis**. Candidates of indole and oxindole compounds are shown as “C.” **(A)** Upper panel, base peak chromatogram (BPC) in the *Catharanthus* roots. Lower panels, extracted ion chromatogram (EIC) at *m/z* 128.04, 144.08, and 204.07 assigned as C_9_H_6_N, C_10_H_10_N, and C_15_H_10_N, respectively. **(B)** Upper panel, BPC in the *Uncaria* hooks. Lower panels, EIC at *m/z* 145.05, 158.058, and 187.08 assigned as C_9_H_7_NO, C_11_H_11_N_2_O, and C_10_H_8_NO, respectively.

The MS/MS boost analysis was finally applied to the MIA-targeted analysis of indole and oxindole compounds using the product ions in crude samples. The medicinal plant *C. roseus* contains many MIAs with excellent pharmaceutical activity (O’Connor and Maresh, 2006). Therefore, Compounds **3** and **4** could be profiled using the *m/z* values in the root parts (**Figure 6A**). In the medicinal plant *U. rhynchophylla*, Compounds **11** and **12** could be profiled using the *m/z* values in the part of the prickle, which is referred to as a hook (**Figure 6B**). Candidates of indole and oxindole compounds (**C1**–**C8**) could be also profiled in both samples. These results suggested that the use of multiple product ions is useful for the MIA-targeted analysis to profile known and unknown indole and oxindole compounds. To further increase the accuracy of the targeted analysis of MIAs containing indole and oxindole skeletons, tracing the precursor and product ions with their on-line UV spectrum (Supplementary Figure S7) may be suitable.

This analysis is important for the development of a chemical assignment strategy in LC-MS-based metabolomics. The use of elemental composition-assigned product ions as expression sequence tags (ESTs; Rudd, 2003) narrows the pool of possible structures of a single candidate structure. ESTs are used in the study of phytochemical genomics to narrow down target genes using a partial sequence and expressed transcripts of the ESTs regarding the gene in a particular organism. The common or specific ESTs among various species stored in databases provide a first indication of the target gene. After the pool of potential target genes has been narrowed, further experiments such as bioinformatics-based EST clustering, sequence assembly, and gene cloning are necessary for conclusive identification of the target gene. Accumulating the information of chemically assigned product ions in databases is useful for not only the chemical assignment of MIAs but also other specialized metabolites. After narrowing down the pool of possible structures, complete structure identification is required using MS and nuclear magnetic resonance.

## Conclusion

Here, we developed a method for boosting the sensitivity in LC–FTICR–MS/MS analysis, which can be applied to the MIA-targeted analysis. This method achieved the identification of major and minor product ions of MIAs, suggesting that it could provide metabolite features that have been missed so far. The discovery of key product ions will promote strategies of the MIA-targeted analysis. Approximately 3,000 MIAs (O’Connor and Maresh, 2006) including analogs, geometric isomers, and stereoisomers have been reported in plants. The use of their elemental composition-assigned product ions is expected to enable the chemical assignment of the substructure and whole structure in a large number of MIAs in LC–MS-based metabolomics.

## Author Contributions

RN designed the study and interpreted the data. RN and H. Tsugawa conducted the product ion analysis. MK and H. Takayama isolated the MIA compounds. RN, MK, and H. Takayama prepared the plant samples. RN and KS wrote the paper. All the authors commented on the manuscript.

## Conflict of Interest Statement

The authors declare that the research was conducted in the absence of any commercial or financial relationships that could be construed as a potential conflict of interest.
