# Supplementary material for: Boosting Sensitivity in Liquid Chromatography–Fourier Transform Ion Cyclotron Resonance–Tandem Mass Spectrometry for Product Ion Analysis of Monoterpene Indole Alkaloids
Source: Front Plant Sci. 2015 Dec 17;6:1127. doi: 10.3389/fpls.2015.01127 (PMC4681812; doi:10.3389/fpls.2015.01127)
Supplement: Supplementary file 1 [file Data_Sheet_1.ZIP › SupplementaryMaterials/SupplementaryMaterials_Figures_revised.pptx]

## Slide 1
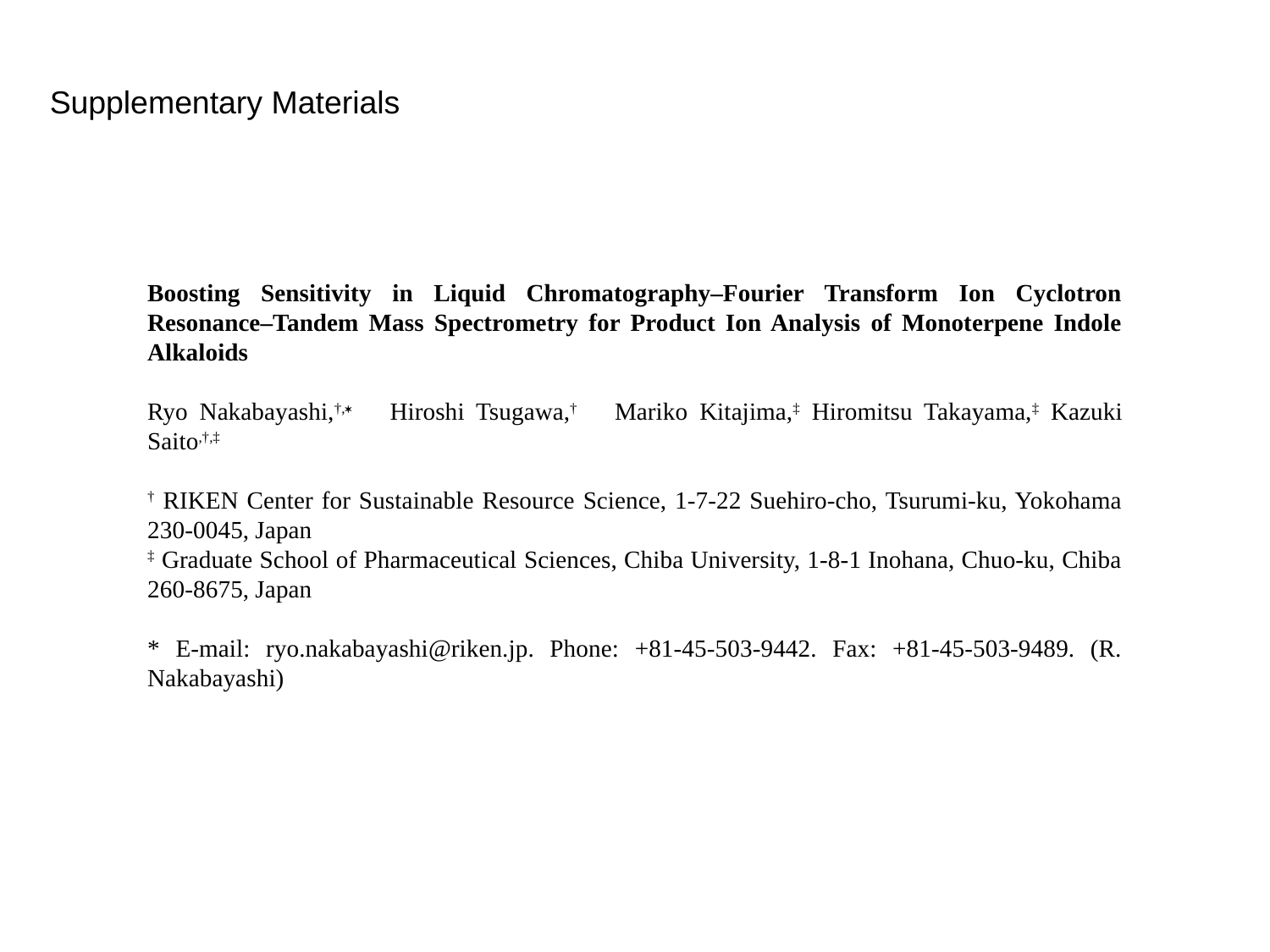

Supplementary Materials
Boosting Sensitivity in Liquid Chromatography–Fourier Transform Ion Cyclotron Resonance–Tandem Mass Spectrometry for Product Ion Analysis of Monoterpene Indole Alkaloids
Ryo Nakabayashi,†,　Hiroshi Tsugawa,†　Mariko Kitajima,‡ Hiromitsu Takayama,‡ Kazuki Saito,†,‡
† RIKEN Center for Sustainable Resource Science, 1-7-22 Suehiro-cho, Tsurumi-ku, Yokohama 230-0045, Japan
‡ Graduate School of Pharmaceutical Sciences, Chiba University, 1-8-1 Inohana, Chuo-ku, Chiba 260-8675, Japan
* E-mail: ryo.nakabayashi@riken.jp. Phone: +81-45-503-9442. Fax: +81-45-503-9489. (R. Nakabayashi)

## Slide 2
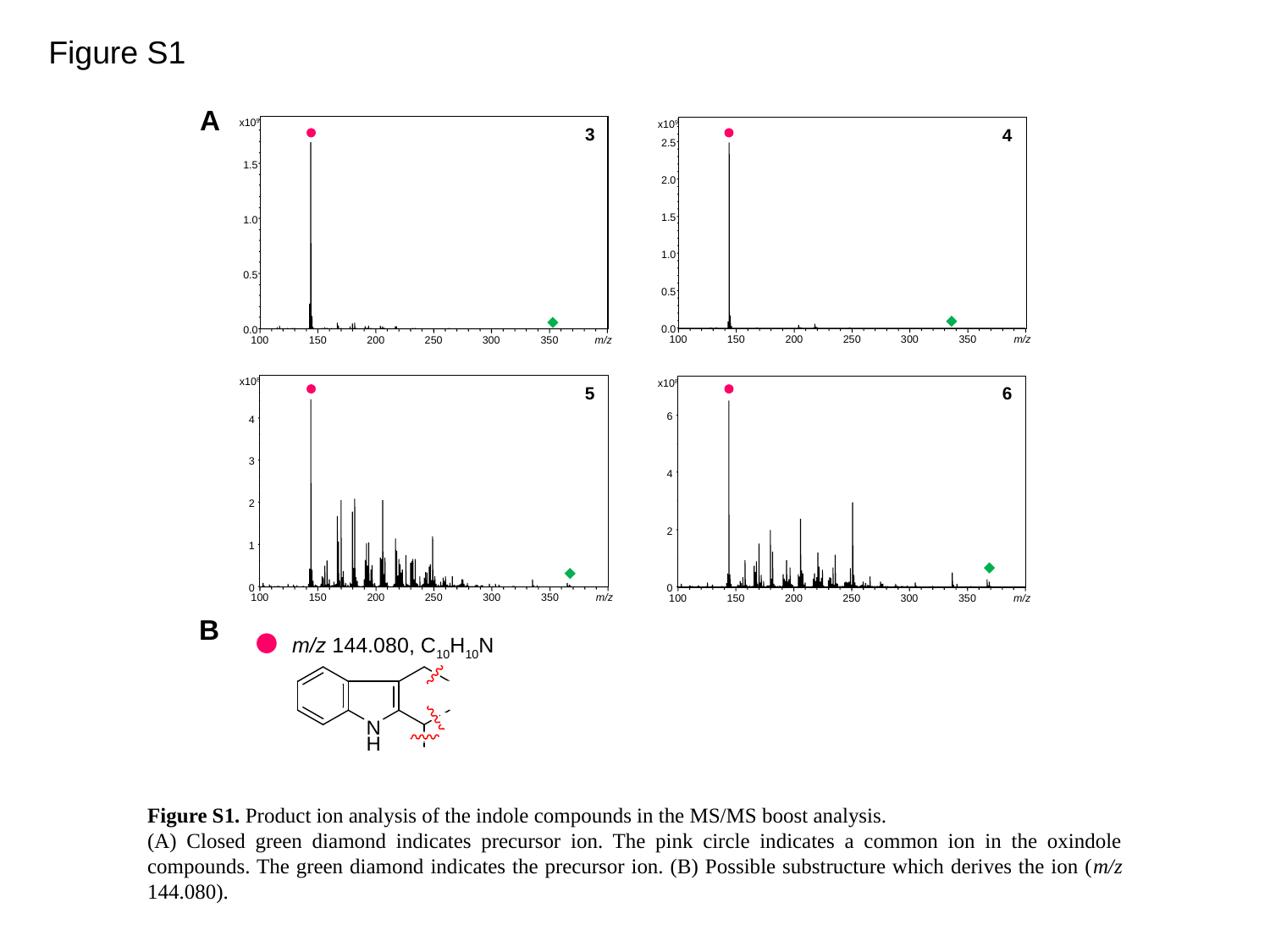

Figure S1
A
x109
1.5
1.0
0.5
0.0
100
150
200
250
300
350
m/z
x109
2.5
2.0
1.5
1.0
0.5
0.0
100
150
200
250
300
350
m/z
x108
4
3
2
1
0
100
150
200
250
300
350
m/z
x108
6
4
2
0
100
150
200
250
300
350
m/z
3
4
5
6
B
m/z 144.080, C10H10N
Figure S1. Product ion analysis of the indole compounds in the MS/MS boost analysis.
(A) Closed green diamond indicates precursor ion. The pink circle indicates a common ion in the oxindole compounds. The green diamond indicates the precursor ion. (B) Possible substructure which derives the ion (m/z 144.080).

## Slide 3
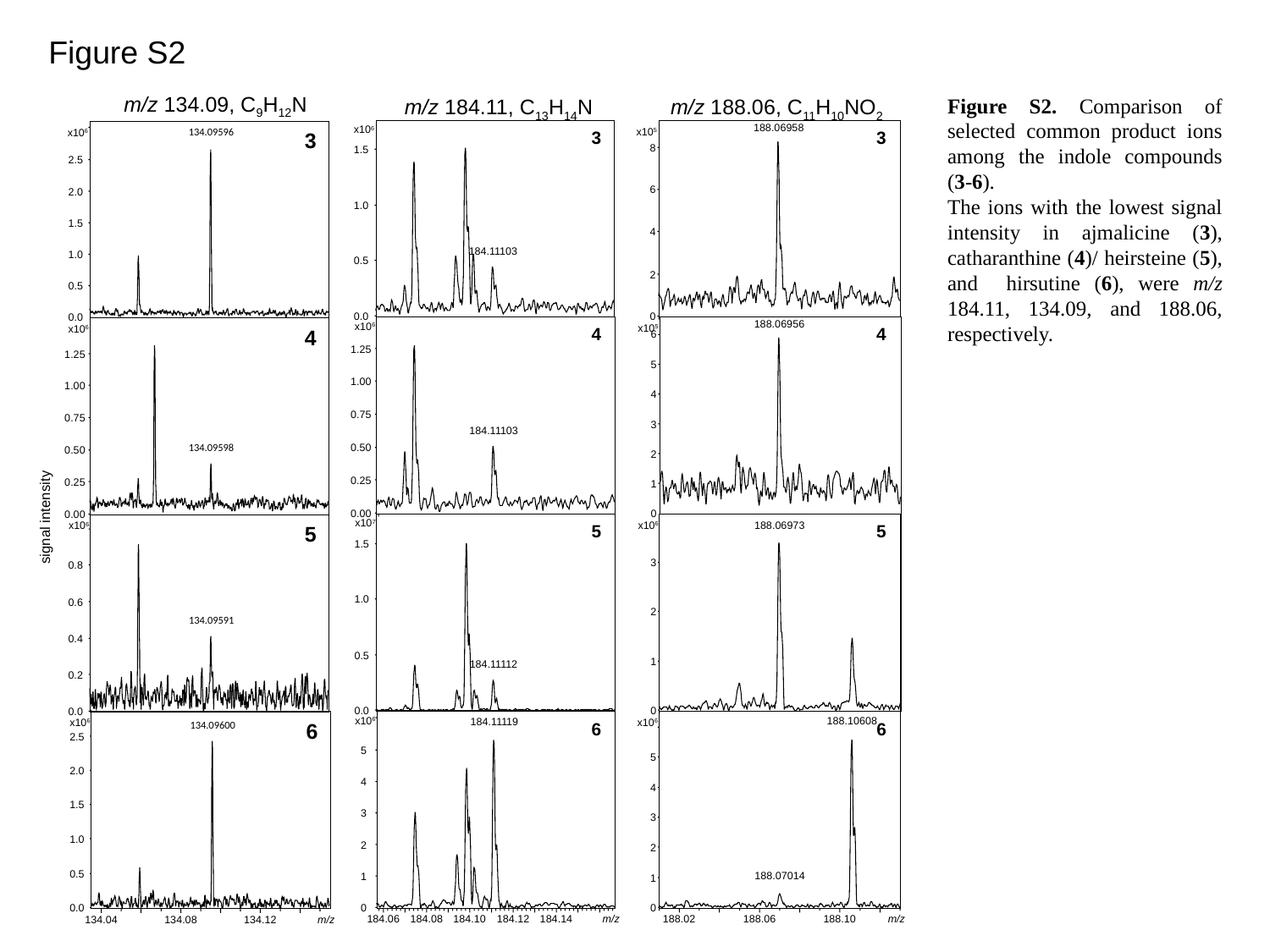

Figure S2
Figure S2. Comparison of selected common product ions among the indole compounds (3-6).
The ions with the lowest signal intensity in ajmalicine (3), catharanthine (4)/ heirsteine (5), and hirsutine (6), were m/z 184.11, 134.09, and 188.06, respectively.
m/z 134.09, C9H12N
3
134.09596
x106
2.5
2.0
1.5
1.0
0.5
4
x106
1.25
1.00
0.75
134.09598
0.50
0.25
0.00
signal intensity
0.0
5
x106
0.8
0.6
134.09591
0.4
0.2
0.0
6
x106
134.09600
2.5
2.0
1.5
1.0
0.5
0.0
134.04
134.08
134.12
m/z
m/z 184.11, C13H14N
3
x106
1.5
1.0
184.11103
0.5
0.0
4
x106
1.25
1.00
0.75
184.11103
0.50
0.25
0.00
5
x107
1.5
1.0
0.5
184.11112
0.0
6
x106
184.11119
5
4
3
2
1
0
184.06
184.08
184.10
184.12
184.14
m/z
m/z 188.06, C11H10NO2
188.06958
3
x105
8
6
4
2
0
4
188.06956
x105
6
5
4
3
2
1
0
5
188.06973
x106
3
2
1
0
6
188.10608
x106
5
4
3
2
188.07014
1
0
188.02
188.06
188.10
m/z

## Slide 4
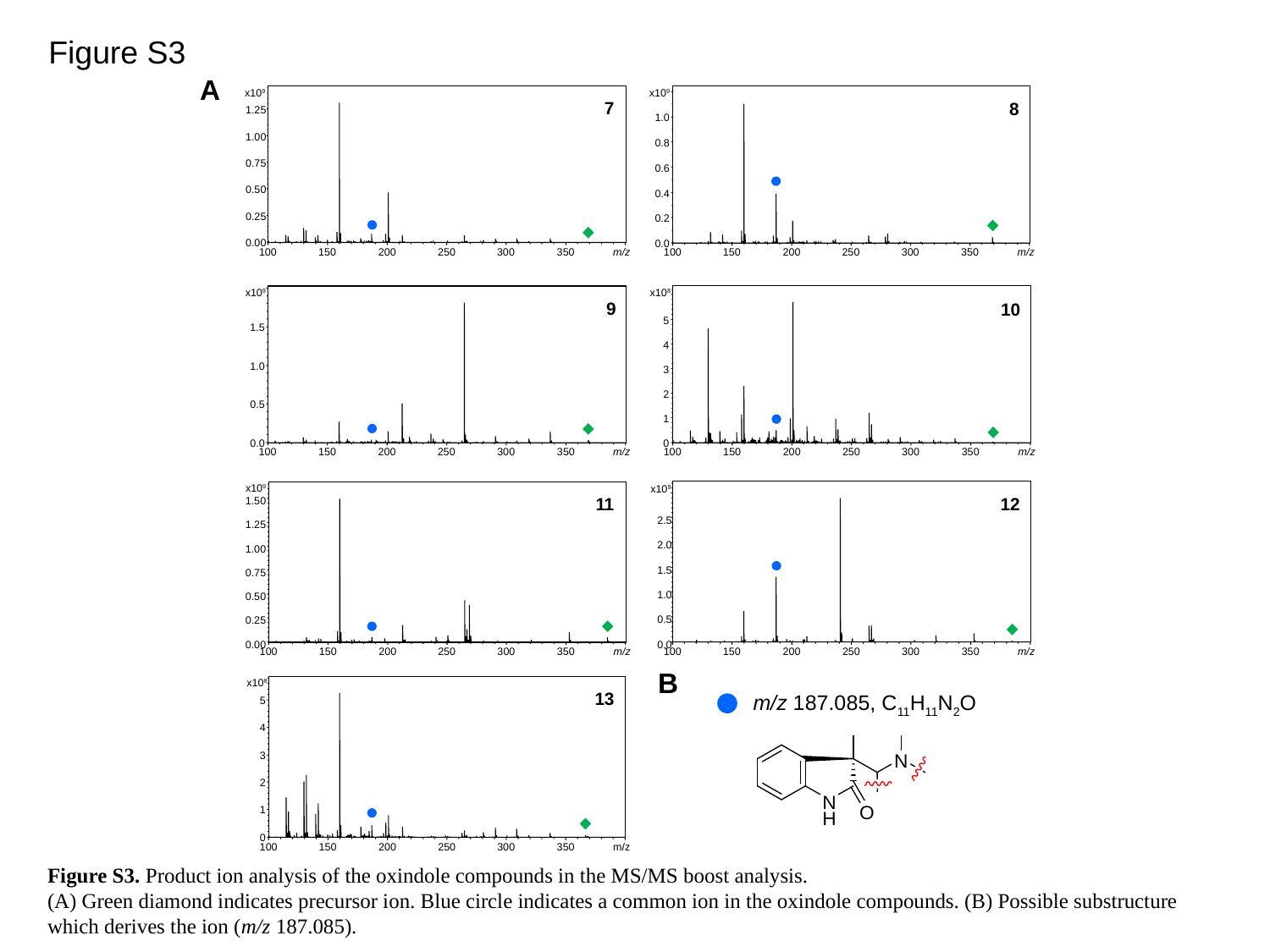

Figure S3
A
x109
1.0
0.8
0.6
0.4
0.2
0.0
100
150
200
250
300
350
m/z
x109
1.25
1.00
0.75
0.50
0.25
0.00
100
m/z
150
200
250
300
350
x108
5
4
3
2
1
0
100
150
200
250
300
350
m/z
x109
1.5
1.0
0.5
0.0
150
200
250
300
350
m/z
100
x109
2.5
2.0
1.5
1.0
0.5
0.0
100
150
200
250
300
350
m/z
x109
1.50
1.25
1.00
0.75
0.50
0.25
0.00
100
150
200
250
300
350
m/z
7
8
9
10
11
12
x108
5
4
3
2
1
0
150
200
250
300
350
m/z
100
13
B
m/z 187.085, C11H11N2O
Figure S3. Product ion analysis of the oxindole compounds in the MS/MS boost analysis.
(A) Green diamond indicates precursor ion. Blue circle indicates a common ion in the oxindole compounds. (B) Possible substructure which derives the ion (m/z 187.085).

## Slide 5
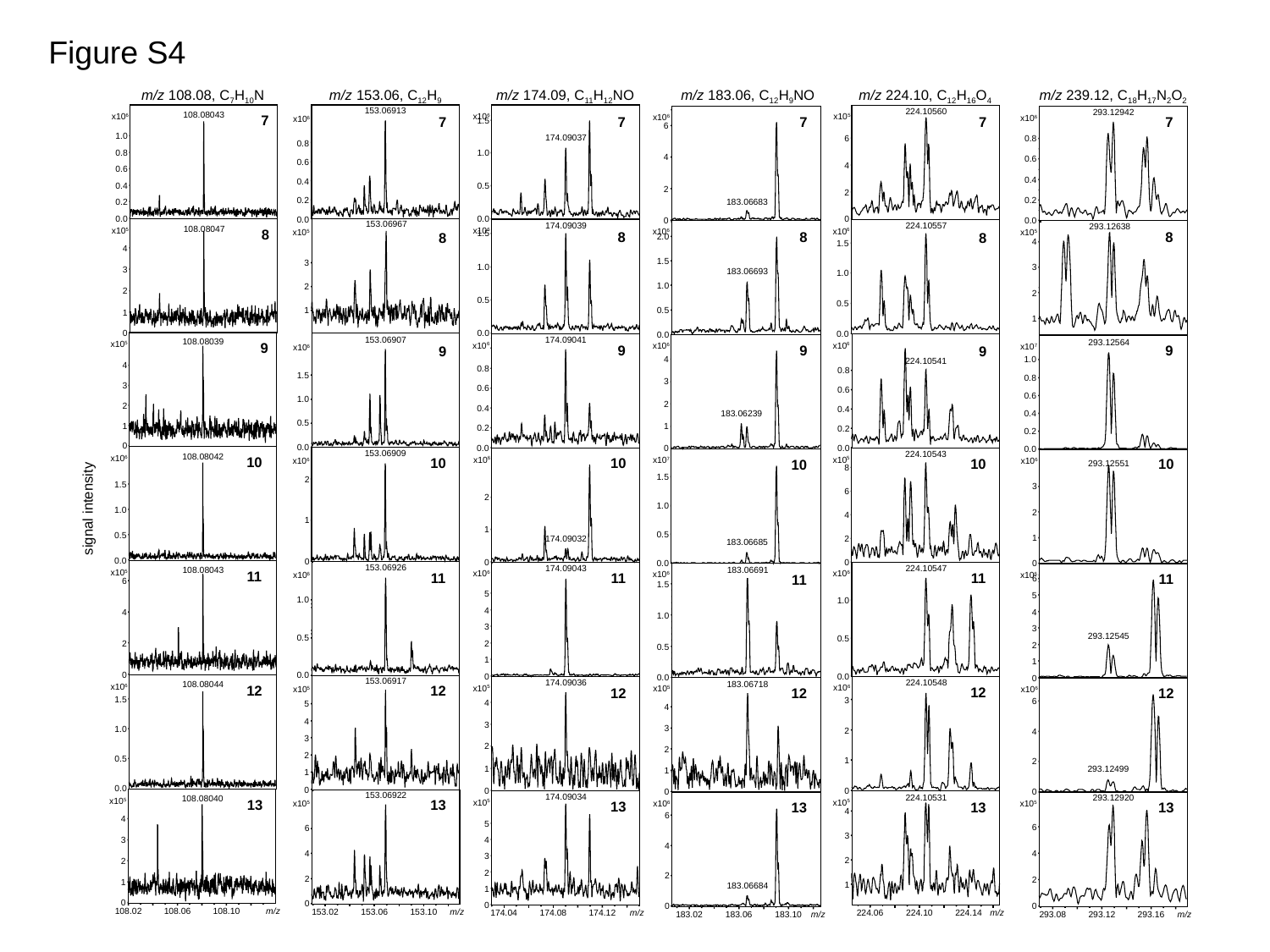

Figure S4
m/z 224.10, C12H16O4
224.10560
7
x105
6
4
2
0
224.10557
8
x106
1.5
1.0
0.5
0.0
9
x106
224.10541
0.8
0.6
0.4
0.2
0.0
224.10543
10
x105
8
6
4
2
0
224.10547
11
x106
1.0
0.5
0.0
224.10548
12
x106
3
2
1
0
224.10531
13
x105
4
3
2
1
224.06
224.10
224.14
m/z
m/z 108.08, C7H10N
m/z 153.06, C12H9
153.06913
7
x106
0.8
0.6
0.4
0.2
0.0
153.06967
8
x105
3
2
1
153.06907
9
x106
1.5
1.0
0.5
0.0
153.06909
10
x106
2
1
0
153.06926
11
x106
1.0
0.5
0.0
12
153.06917
x105
5
4
3
2
1
0
153.06922
13
x105
6
4
2
0
153.02
153.06
153.10
m/z
m/z 174.09, C11H12NO
7
x106
1.5
174.09037
1.0
0.5
0.0
174.09039
8
x106
1.5
1.0
0.5
0.0
174.09041
9
x106
0.8
0.6
0.4
0.2
0.0
10
x106
2
1
174.09032
0
174.09043
11
x106
5
4
3
2
1
0
174.09036
12
x105
4
3
2
1
0
174.09034
13
x105
5
4
3
2
1
0
174.04
174.08
174.12
m/z
m/z 183.06, C12H9NO
7
x106
x106
x106
x107
x106
x105
x106
6
4
2
183.06683
0
8
2.0
1.5
183.06693
1.0
0.5
0.0
9
4
3
2
183.06239
1
0
10
1.5
1.0
0.5
183.06685
0.0
183.06691
11
1.5
1.0
0.5
0.0
183.06718
12
4
3
2
1
0
13
6
4
2
183.06684
0
183.02
183.06
183.10
m/z
m/z 239.12, C18H17N2O2
293.12942
7
x106
0.8
0.6
0.4
0.2
0.0
293.12638
8
x105
4
3
2
1
9
293.12564
x107
1.0
0.8
0.6
0.4
0.2
0.0
10
x106
293.12551
3
2
1
0
11
x106
6
5
4
3
293.12545
2
1
0
12
x106
6
4
2
293.12499
0
13
293.12920
x105
6
4
2
0
293.08
293.12
293.16
m/z
7
108.08043
x106
1.0
0.8
0.6
0.4
0.2
0.0
8
108.08047
x105
4
3
2
1
0
9
108.08039
x105
4
3
2
1
0
10
108.08042
x106
1.5
1.0
0.5
0.0
11
108.08043
x105
6
4
2
0
12
108.08044
x106
1.5
1.0
0.5
0.0
13
108.08040
x105
4
3
2
1
0
108.02
108.06
108.10
m/z
signal intensity

## Slide 6
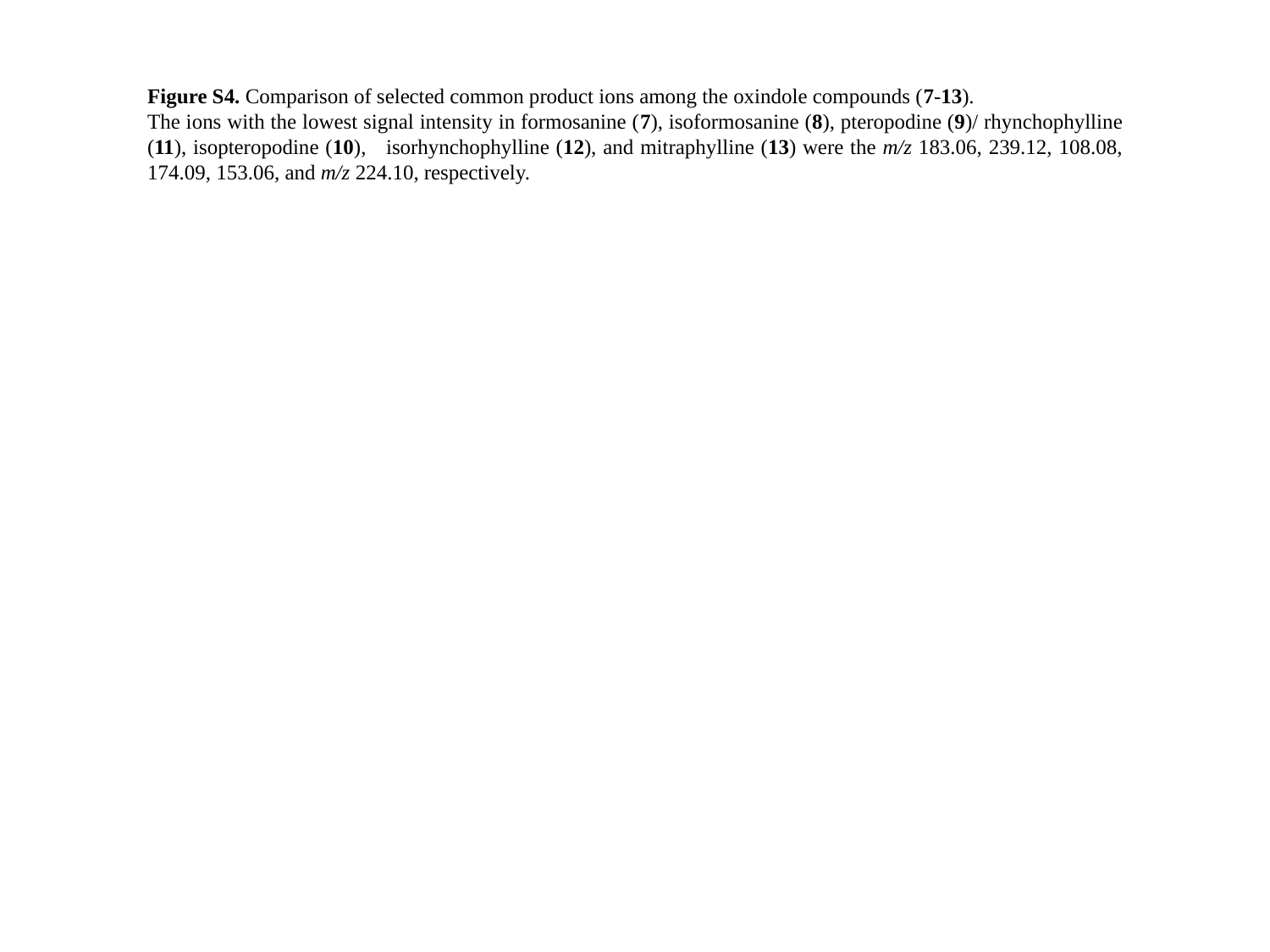

Figure S4. Comparison of selected common product ions among the oxindole compounds (7-13).
The ions with the lowest signal intensity in formosanine (7), isoformosanine (8), pteropodine (9)/ rhynchophylline (11), isopteropodine (10), isorhynchophylline (12), and mitraphylline (13) were the m/z 183.06, 239.12, 108.08, 174.09, 153.06, and m/z 224.10, respectively.

## Slide 7
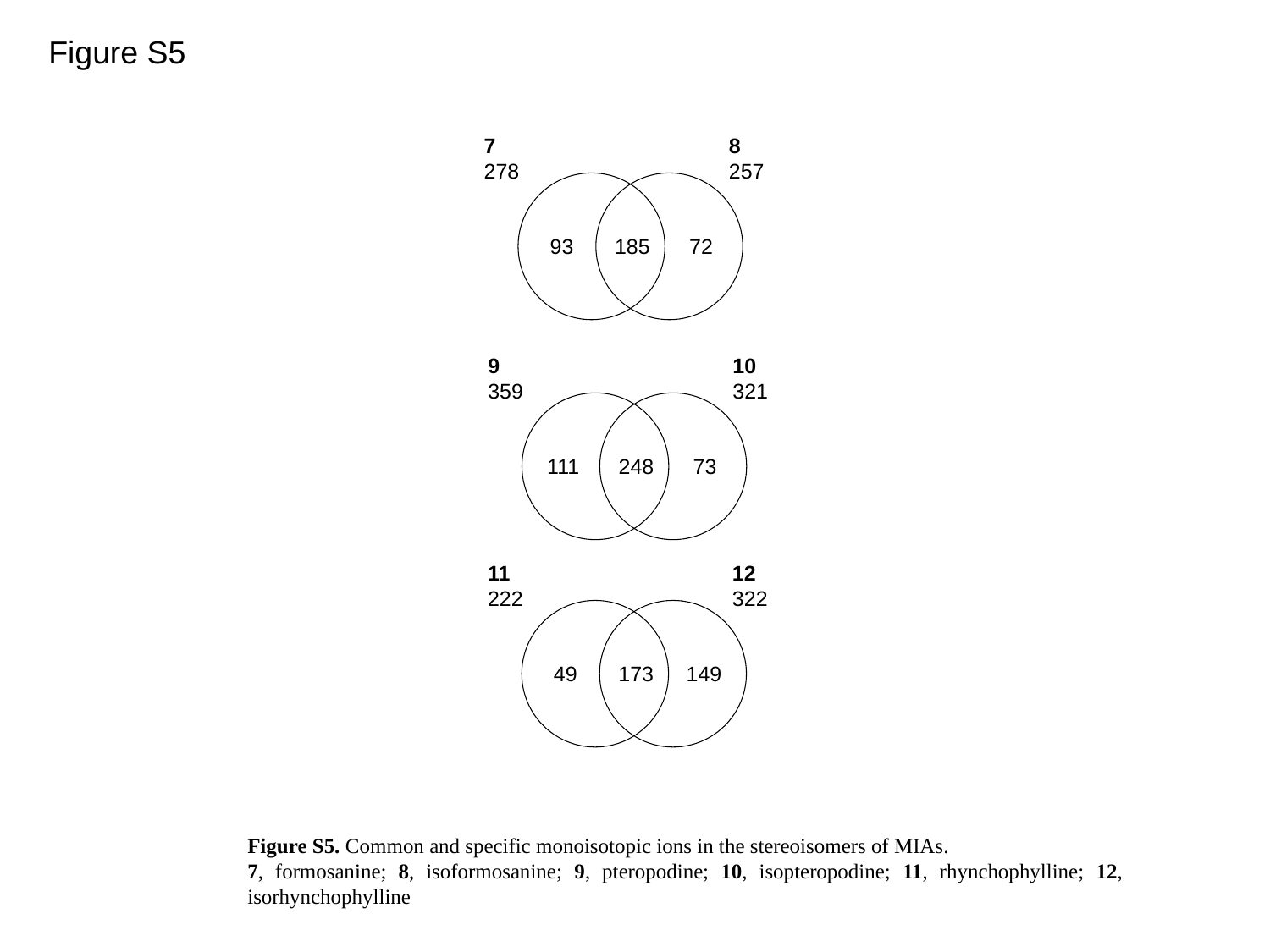

Figure S5
7
278
8
257
93
185
72
9
359
10
321
111
248
73
11
222
12
322
49
173
149
Figure S5. Common and specific monoisotopic ions in the stereoisomers of MIAs.
7, formosanine; 8, isoformosanine; 9, pteropodine; 10, isopteropodine; 11, rhynchophylline; 12, isorhynchophylline

## Slide 8
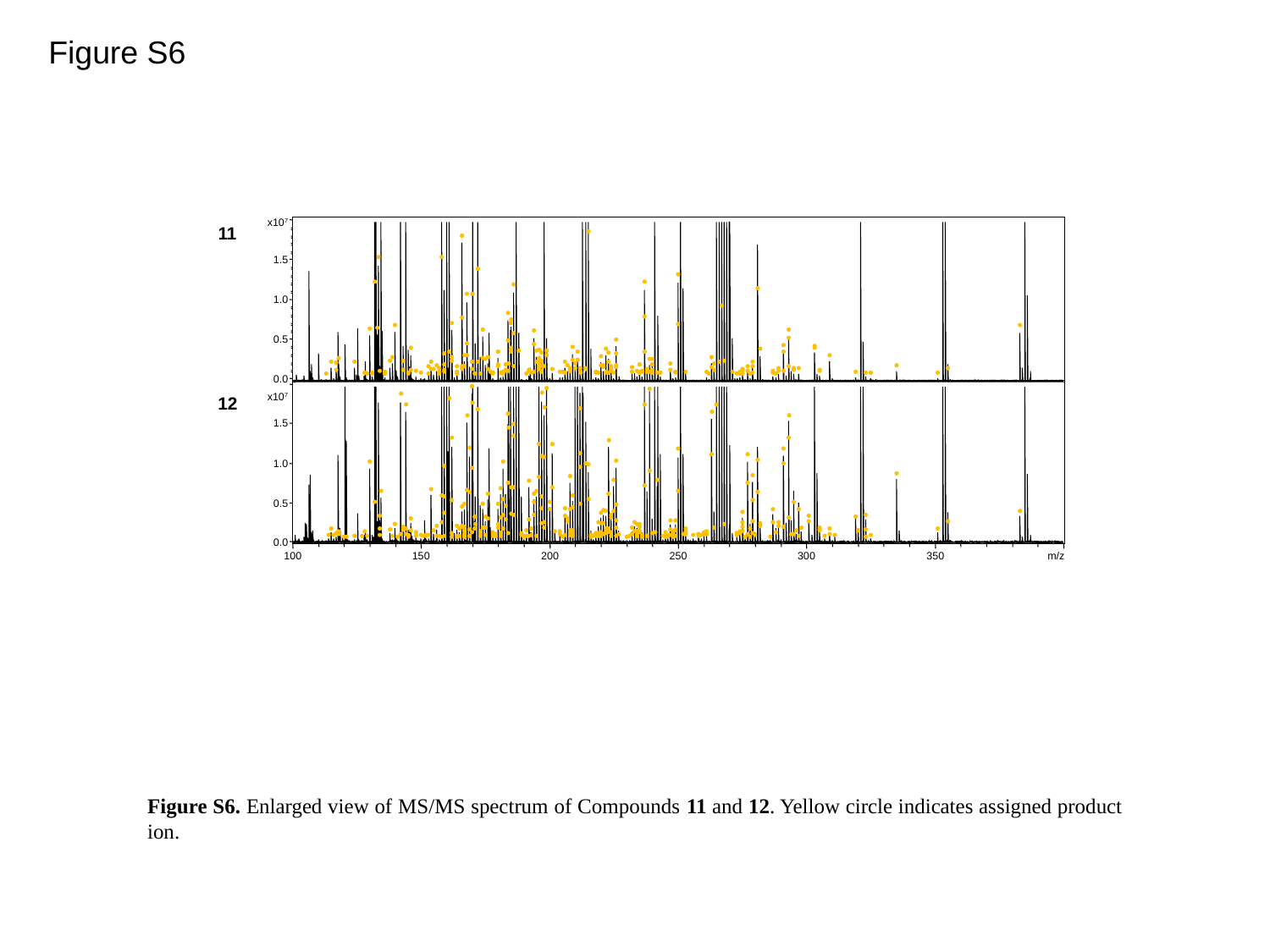

Figure S6
11
x107
1.5
1.0
0.5
0.0
12
x107
1.5
1.0
0.5
0.0
100
150
200
250
300
350
m/z
Figure S6. Enlarged view of MS/MS spectrum of Compounds 11 and 12. Yellow circle indicates assigned product ion.

## Slide 9
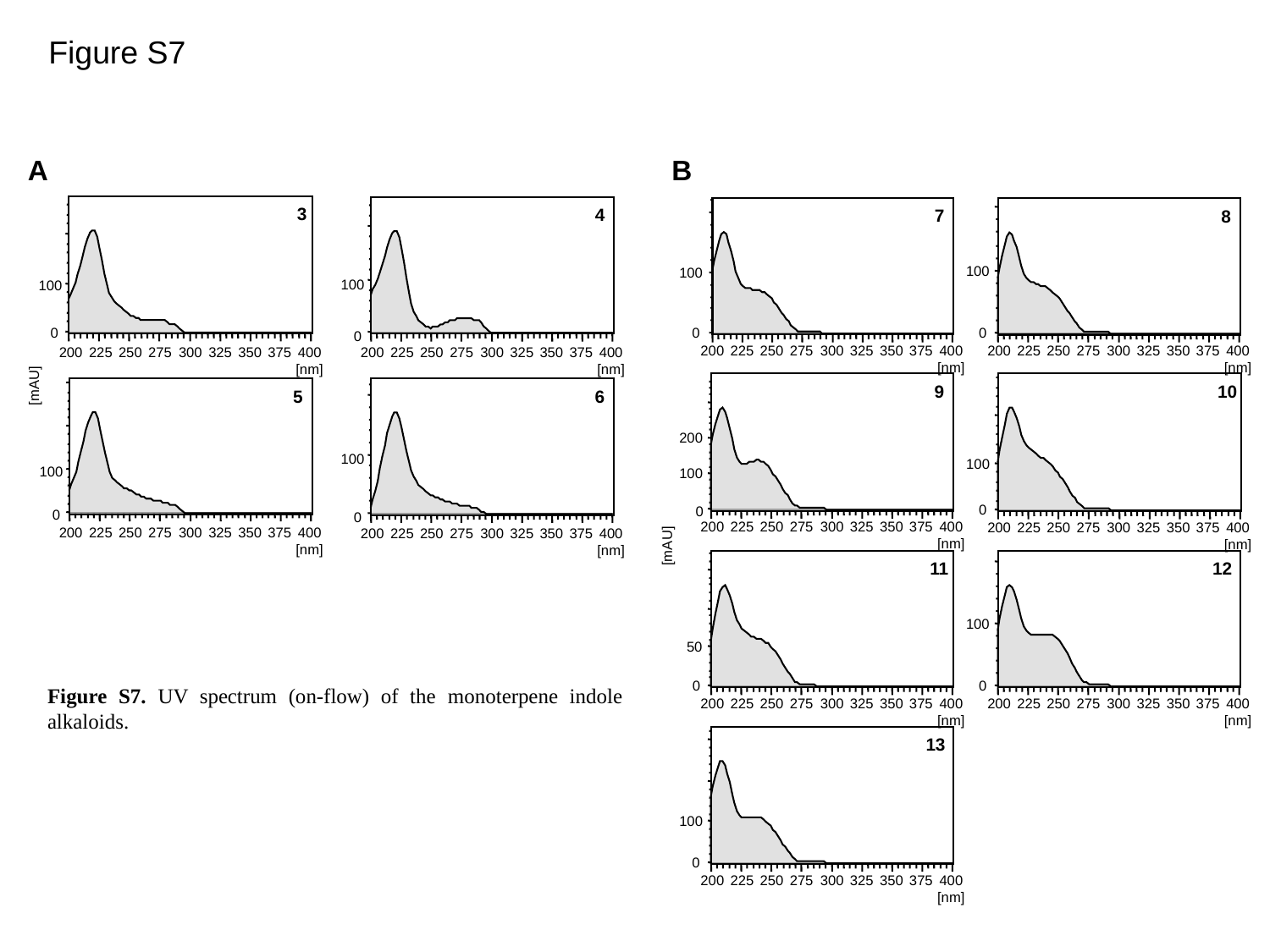

Figure S7
A
B
3
100
0
200
225
250
275
300
325
350
375
400
[nm]
4
100
0
200
225
250
275
300
325
350
375
400
[nm]
6
100
0
200
225
250
275
300
325
350
400
[nm]
375
5
100
0
200
225
250
275
300
325
350
400
[nm]
375
7
100
0
200
225
250
275
300
325
350
375
400
[nm]
9
200
100
0
200
225
250
275
300
325
350
375
400
[nm]
11
50
0
200
225
250
275
300
325
350
375
400
[nm]
13
100
0
200
225
250
275
300
325
350
375
400
[nm]
8
100
0
200
225
250
275
300
325
350
375
400
[nm]
10
100
0
200
225
250
275
300
325
350
375
400
[nm]
12
100
0
200
225
250
275
300
325
350
375
400
[nm]
[mAU]
[mAU]
Figure S7. UV spectrum (on-flow) of the monoterpene indole alkaloids.
